# Oral cenesthopathy

**DOI:** 10.1186/s13030-016-0071-7

**Published:** 2016-06-10

**Authors:** Yojiro Umezaki, Anna Miura, Motoko Watanabe, Miho Takenoshita, Akihito Uezato, Akira Toriihara, Toru Nishikawa, Akira Toyofuku

**Affiliations:** Psychosomatic Dentistry Clinic, Dental Hospital, Tokyo Medical and Dental University, Bunkyo, Japan; Department of Psychosomatic Dentistry, Graduate School of Medical and Dental Sciences, Tokyo Medical and Dental University, Bunkyo, Japan; Department of Psychiatry and Behavioral Sciences, Graduate School of Medical and Dental Sciences, Tokyo Medical and Dental University, Bunkyo, Japan; Department of Diagnostic Radiology and Nuclear Medicine, Graduate School of Medical and Dental Sciences, Tokyo Medical and Dental University, Bunkyo, Japan

**Keywords:** Oral cenesthopathy, Oral dysesthesia, Abnormal bodily sensation, Delusional disorder somatic type

## Abstract

Cenesthopathy is characterized by abnormal and strange bodily sensations and is classified as a ‘delusional disorder, somatic type’ or ‘somatoform disorder’ according to the DSM 5. The oral cavity is one of the frequent sites of cenesthopathy, thus the term ‘oral cenesthopathy.’ Patients with oral cenesthopathy complain of unusual sensations without corresponding abnormal findings in the oral area, such as excessive mucus secretion, a slimy sensation, or a feeling of coils or wires being present within the oral region. They usually visit multiple dentists rather than psychiatrists. Without a proper diagnosis, they repeatedly pursue unnecessary surgical procedures to remove their ‘foreign body’. This sometimes creates a dilemma between the dentists and patients. The nosography of oral cenesthopathy has been discussed in some case reports and reviews but is overlooked in mainstream medicine. This review focuses on the various aspects of oral cenesthopathy.

The estimated prevalence of cenesthopathy was 0.2 to 1.9 % in a study done at a Japanese university psychiatry clinic and 27 % in a study done at a Japanese psychosomatic dentistry clinic. Oral cenesthopathy do not have clear disposition, while some studies reported that elderly women were most commonly affected. Its pathophysiology has not been fully elucidated. However, recent studies have suggested a right > left asymmetrical pattern of the cerebral blood flow of patients with oral cenesthopathy. Antidepressants, antipsychotic drugs, electroconvulsive therapy, and psychotherapy might be effective in some cases, though it is known to be intractable.

To date, the epidemiology, pathophysiology, etiology, classification and treatment of oral cenesthopathy are unknown due to the few reports on the disorder, though there are a few case reports. To overcome this difficult medical condition, clinico-statistical and case–control studies done under rigorous criteria and with a large sample size are required.

## Background

Cenesthopathy is characterized by abnormal and strange bodily sensations [1, 2] and is defined as any localized distortion of body awareness [3]. The oral cavity is one of the frequent sites of cenesthopathy, thus the term ‘oral cenesthopathy.’ Patients with oral cenesthopathy complain of unusual sensations without corresponding abnormal findings in the oral area, such as excessive mucus secretion, a slimy sensation, a squeezing-pulling sensation in the mouth, or a feeling of coils or wires being present within the oral region.

Cenesthopathy was first proposed as a clinical entity by Dupré and Camus in 1907 [1]; however, it long gained little attention. Huber et al. described cenesthetic schizophrenia in 1957 [4]. In Japan, Hozaki et al. reported five patients with chronic cenesthesic hallucination in 1959 [5], and four of the patients complained of abnormal sensations in the mouth.

According to the Diagnostic and Statistical Manual of Mental Disorders, Fifth edition (DSM 5) [6], oral cenesthopathy is classified as a ‘delusional disorder, somatic type (DDST)’. In the ICD-10 Classification of Mental and Behavioral Disorders (ICD-10) [7], it is categorized as a ‘persistent delusional disorder’ or ‘other schizophrenia. The diagnosis of oral cenesthopathy is still controversial, and contemporary medicine does not provide independently defined diagnostic criteria.

Because dental treatment (such as prosthesis or tooth extraction) could be a trigger of oral cenesthopathy, the patients tend to insist on further dental treatment. Hence, the dentists are sometimes troubled by the management of such treatments. On the other hand, in the field of psychiatry, though the relevance has been indicated between oral cenesthopathy and schizophrenia or depression, it does not necessarily occur as a part of the symptoms of these psychiatric disorders. In addition, these symptoms rarely react to specific medications. Because in many cases the patients do not show any other psychiatric symptoms, some psychiatrists are of the opinion that they do not need to see patients with oral cenesthopathy [8]. Moreover, because the patients with oral cenesthopathy generally do not seek psychiatric consultation on their own but rely on dental examination, the mental and dental collaboration is very important [9].

The nosography of oral cenesthopathy has been discussed in some case reports and reviews but has been overlooked in mainstream medicine [10]. The present review focuses on various aspects of oral cenesthopathy (including delusional disorder, which seems to be oral cenesthopathy according to the definition), such as its epidemiology, pathophysiology, etiology, clinical presentation, and current treatment.

## Epidemiology

The prevalence of oral cenesthopathy is unknown due to the lack of strict epidemical studies. To the best of our knowledge, only three Japanese papers have reported the estimated prevalence of cenesthopathy. Wake et al. [11] reported that 18 out of 10,278 outpatient cases (0.175 %) seen in 5.5 years were diagnosed as cenesthopathy in a University Psychiatry Clinic in Okayama, Japan. Yoshimatsu [12] reported that 31 out of 1670 inpatient cases (1.86 %) in 12 years and 37 out of 15,600 outpatient cases (0.24 %) in 3 years were diagnosed as cenesthopathy in a University Psychiatry Clinic in Tokyo, Japan. Among these cenesthopathy patients, around 85 % were reported to have oral cenesthopathy [13]. In the psychosomatic dentistry clinic in a Japanese dental hospital, 332 out of 1210 outpatient cases (27.44 %) in 3 years were diagnosed as oral cenesthopathy [14].

Oral cenesthopathy does not have a clear gender or age predisposition. Some researchers reported that the age distribution is bimodal, 20’s to 30’s and 50’s [12, 15]. Another study [16] showed that oral cenesthopathy was predominant in elderly female patients and that non-oral cenesthopathy was predominant in younger male patients.

## Pathophysiology

The pathophysiology of oral cenesthopathy has not been fully elucidated. In some cases, organic causes of oral cenesthopathy are indicated. For example, one case report described a patient who developed oral cenesthopathy resulting from direct damage to the thalamocortical tract [17], while another report discussed a stroke patient with oral cenesthopathy due to cortical reorganization [18]. However, in these case reports, no cerebrovascular disease corresponding oral cenesthopathy was indicated using computed tomography (CT) [19] or structural magnetic resonance imaging (MRI) [20].

**Fig. 1:**
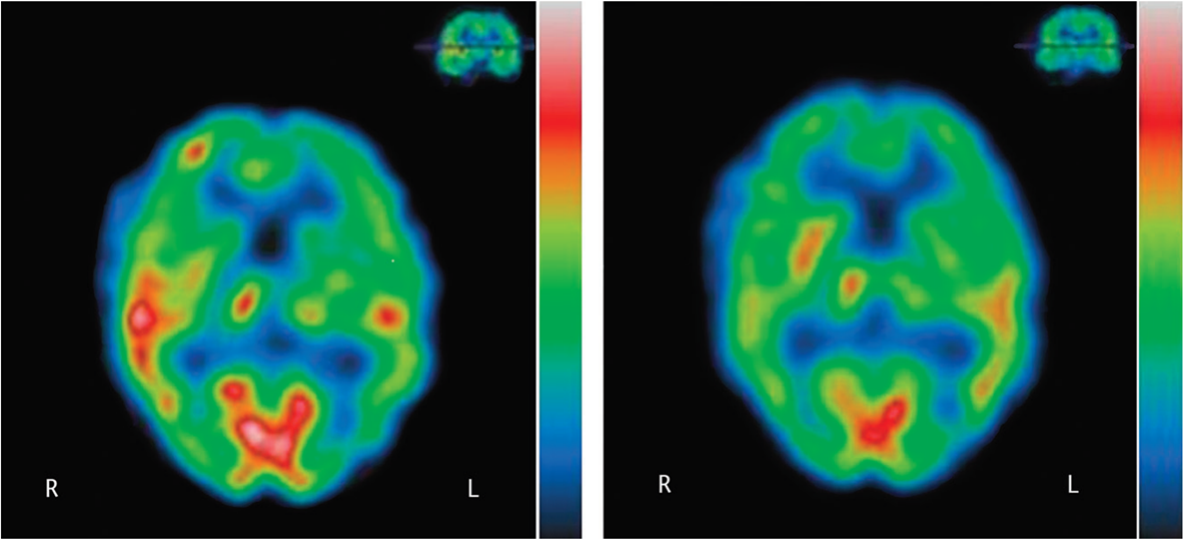
Single photon emission computed tomography (technetium-99 m-ethyl cysteinate dimer) images before (*left*) and after (*right*) treatment [24]. Hyperperfusion in the right relative to the left temporal lobe improved after treatment in parallel with the alleviation of the oral cenesthopathy

Some functional brain imaging studies have also been reported. Tateno et al. [21] reported that patients with oral cenesthopathy showed significantly higher regional cerebral blood flow (rCBF) in the right anterior cingulate and bilateral thalamus than did patients with depression using Iodine-123-iodoamphetamine (^123^I-IMP) single photon emission computed tomography (SPECT). We recently reported a right > left asymmetrical rCBF pattern in a broad area of the brain, including the frontal and temporal lobes of patients with oral cenesthopathy as compared to healthy control subjects using technetium-99 m-ethyl cysteinate dimer (^99m^Tc-ECD) SPECT [22]. The right > left asymmetrical rCBF pattern was also confirmed in another study among patients with oral cenesthopathy, with or without depression, while the mean rCBF value of patients in the depression group were lower in several brain regions [23]. A case report showed that the condition of a patient with oral cenesthopathy and hyperperfusion in the right relative to the left temporal lobe improved after modified electroconvulsive therapy (mECT), in parallel with the alleviation of the clinical symptoms [24] (Fig. 1). In terms of DDST, some rCBF studies have reported improvement in decreased rCBF in the left temporal and parietal regions after successful treatment [25–27]. In these studies and case reports, right-side-predominant rCBF asymmetry is consistent. On the other hand, Nemoto et al. [28] reported that patients with DDST (in 4 of the 5 patients the delusional symptoms were localized in the oral area) exhibited a significant increase in perfusion in the left post-central gyrus and right paracentral lobule.

As became evident from these studies, the pathophysiology of oral cenesthopathy is highly complex, and the complicated clinical entity makes it difficult to study. The heterogeneousness of the rCBF pattern suggests that various subgroups may have been included in the clinical entity of oral cenesthopathy. Moreover, brain imaging study done using rigorous criteria is needed to reveal the pathophysiology of oral cenesthopathy.

## Etiology

The exact etiology of oral cenesthopathy remains imprecise and controversial. Though the relationship between cenesthopathy and psychiatric disorder has been reported in many papers, it is well known that cenesthopathy is developed even without any psychiatric disorders. As to personality characteristics, a case control study pointed out that patients with oral cenesthopathy tended to be socially immature and that they are inept at responding to a Rorschach test [29].

Many case reports [11, 13, 18, 30–36] have reported that some dental treatments, including tooth extraction, periodontal surgery, and denture treatment, can be the trigger of oral cenesthopathy. Thus, it is presumed that some change in the oral environment might be a critical factor in the development of oral cenesthopathy. Additionally, organic brain change [17, 18] and toxic factors [37] have also been considered as critical factors.

## Clinical presentation and assessment tool

Patients with oral cenesthopathy complain of many kinds of abnormal oral sensation without corresponding abnormal findings, as exemplified in Table 1. We recently developed the Oral Dysesthesia Rating Scale (Oral DRS; http://www.tmd.ac.jp/med/psyc/research/oral-drs.html) [10] to reorganize and objectify the complicated symptoms of oral cenesthopathy. In the Oral DRS, a Symptom Severity Scale (SSS) [A], a Functional Impairment Scale (FIS) [B], and a Visual Analog Scale (VAS) [C] are evaluated through semi-structured interviews. The SSS [A] consists of seven categories: feeling a foreign body [A1], exudation [A2], squeezing-pulling [A3], movement [A4], misalignment [A5], pain [A6], and spontaneous thermal sensation or tastes [A7]. The FIS [B] evaluates the severity of impairment of eating [B1], articulation [B2], work [B3], and social activities [B4]. The VAS [C] assesses the overall subjective severity of the symptoms [C1] and changes in the severity of the symptoms [C2].

**Table 1:** Examples of oral cenesthopathy complaints

"A wire is coming out of my gum”.	
"There are coils around my teeth”.	
"Something slimy is always in my mouth”.	
"Gas is blowing up in my teeth”.	
"Something like a thread is coming out from between my teeth”.	
"I have a squeezing sensation in my mouth”.	
"My gum is twisting”.	
"Excessive saliva and bubbles are in my mouth”.	

### Classification

The classification of oral cenesthopathy is ambiguous in contemporary medicine [38]. In DSM 5, cenesthopathy is categorized under DDST. However, this clinical category (i.e., DDST) may involve certain issues. The somatic type of delusional disorder is the only one that was not included in Kraepelin’s original description of paranoia. It is diagnosed when the central themes of the delusional system are of a hypochondriacal or somatic nature [39]. According to this definition, various previously described diseases, such as cenesthopathy, delusional parasitosis, bromosis, and monosymptomatic hypochondriasis (or monosymptomatic hypochondriacal psychosis), are classified together into DDST in DSM 5. This diagnostic problem and unknown etiology suggest that various clinical states may be put together into a single clinical entity, DDST.

The etiology of oral cenesthopathy is still unknown, but the symptoms are clearly caused and exacerbated by psychological factors. However, it does not have any corresponding organic disorder. Hence the definition of psychosomatic disease is not well applicable to oral cenesthopathy. To answer the precise clinical points that distinguish oral cenesthopathy from other psychosomatic diseases, more research will be needed.

As described above, oral cenesthopathy itself may also have many subtypes. Two classification schemes for cenesthopathy have been proposed, based on either etiology or clinical symptoms. Hozaki [15] classified cenesthopathy into two groups, primary and secondary, based on the etiology. The former is monosymptomatic, and the latter is the one that appears secondary to a psychiatric disorder such as schizophrenia or depression [21, 23]. Yoshimatsu [12] classified it into five groups based on the features of mental manifestation, details of complaint, and attitude. The first group is related to disruption of self-consciousness or depersonalization. The second group is related to a slight sickness. The patients in the third group complain of grotesque and bizarre sensations as if they are real experiences. The attitude of the fourth group is selfish, and their complaints are exaggerated. The fifth group includes others. These five groups are not divided clearly but can be considered as a spectrum classification. However, these classification schemes are currently just conceptual. Hence in the future, a new classification system based on the pathophysiology will be required for clinical application.

### Treatment

The management of oral cenesthopathy remains elusive despite attempts with different classes of medication. The strategies investigated include antidepressants, anti-psychotic drugs, electroconvulsive therapy (ECT), and psychotherapy.

In the case of antidepressants, the efficacies of amitriptyline [17, 33], milnacipran [40], paroxetine [25, 41] and mianserin [19] have been reported. Of the antipsychotic drugs, haloperidol [19, 30], pimozide [13, 20], tiapride [11], sulpiride [17], risperidone [42], perospirone [41, 43] and aripiprazole [44, 45] were reported to be effective for oral cenesthopathy. In addition, the efficacies of lithium carbonate [43] and donepezil [46] have also been reported. Other than pharmacotherapy, the efficacies of ECT [24, 47, 48] and psychotherapy [34–36, 49] have been reported in various studies. However, in some studies, the treatments did not result in any change in the symptoms of oral cenesthopathy [18, 37]. The response rate to various treatments is speculated to be lower than 50 % [13]. Oral cenesthopathy remains an intractable disorder, and further research is needed to find new methods to manage it.

## Conclusion

Oral cenesthopathy is a strange oral sensation without corresponding abnormal findings. The patients usually visit multiple dentists, rather than psychiatrists, seeking invasive treatment and repeatedly try unnecessary procedures. This may sometimes create a dilemma between the dentists and patients.

To date, the epidemiology, pathophysiology, etiology, classification, and treatment of oral cenesthopathy are unknown: few reports on the disorder have been done, though there are a few case reports. To overcome this difficult medical condition, further clinico-statistical and case–control studies that use rigorous criteria and that have a large sample size will be required.

## Abbreviations

^123^I-IMP, Iodine-123-iodoamphetamine; ^99m^Tc-ECD, technetium-99 m-ethyl cysteinate dimer; CT, computed tomography; DDST, delusional disorder, somatic type; DSM 5, Diagnostic and Statistical Manual of Mental Disorders, Fifth edition; ECT, electroconvulsive therapy; ICD-10, ICD-10 Classification of Mental and Behavioral Disorders; mECT, modified electroconvulsive therapy; MRI, magnetic resonance imaging; Oral DRS, Oral Dysesthesia Rating Scale; rCBF, regional cerebral blood flow; SPECT, single photon emission computed tomography.
